# The Road to a Hypoglycemic Care Model

**DOI:** 10.7759/cureus.5865

**Published:** 2019-10-08

**Authors:** Markus Moore, Saeed K Alzghari

**Affiliations:** 1 Miscellaneous, College of Pharmacy, University of North Texas Health Science Center, Fort Worth, USA; 2 Pharmacy, Baylor Scott & White Medical Center, Waxahachie, USA

**Keywords:** hypoglycemia, inpatient, diabetes, models of care, glycemic control

## Abstract

Hypoglycemia is a modifiable condition that causes mortality and readmission rates to increase in a hospital setting. The condition is worsened by its complications, quick onset, and an absence of a protocol mapped out by hospitals. This complication does not come from one origin, but rather from a range of barriers. Research from a variety of sources, such as those found from the American Diabetes Association, have shown great promises in ways to treat and prevent hypoglycemia that are of importance to highlight for practitioners in the inpatient setting.

## Editorial

Hypoglycemia is a condition wherein a person’s blood glucose level is below 70 mg/dL. The signs and symptoms of this disorder can range from feeling anxious or lightheaded to more severe indicators such as unconsciousness or even seizures. Due to its quick onset and the way an individual may experience the signs and symptoms of low blood glucose, hypoglycemia can turn deadly to those without proper treatment. Mortality and readmission rates are the main concern for patients being discharged with hypoglycemia. The issue that arises with hypoglycemia is that it is a modifiable variable that has increased mortality and readmission rates [1]. With effective end-of-stay care, patient education tools, or staff re-education, the trend can move toward the opposite direction.

The prevalence of hypoglycemia is commonly found with individuals who have type 1 diabetes mellitus, especially in the elderly population. This condition is more likely caused by those who take insulin; however, other factors may cause hypoglycemia such as missing a meal, taking certain classes of anti-diabetic medications (i.e. sulfonylureas such as glipizide or glimepiride), or consuming excessive amounts of alcohol. It is important for individuals to always monitor and record their blood glucose levels regularly to make sure it is within their target range to prevent hypoglycemia.

Patients who were hospitalized due to their complications of diabetes have shown that hypoglycemia is associated with an increase in the length of stay, mortality risk, and early readmission to an inpatient hospital stay. One study found that those suffering from a mild form of hypoglycemia have shown a 60% increase in inpatient mortality rate while patients with severe hypoglycemia had a 105% increase [2]. Furthermore, physicians should aim to keep the patient's blood glucose levels to at least 138 mg/dL to avoid possible hypoglycemic events [2]. With these goals in mind, patients can have improved outcomes once discharged from an inpatient setting.

A recent study was conducted by the Veteran Affairs hospital over the course of 14 years, with nearly 844,000 admissions and came to the conclusion that within a 30-day period, both post-discharge mortality and 30-day readmission rates were higher if a patient experienced hypoglycemia or glucose levels near the hypoglycemia threshold [3]. While this study had some limitations, they concluded that additional investigation will be needed to ensure optimal care for patients within 24 hours of being discharged.

Although there is no universal proven way to solve the dilemma described above, there are some tools to help prevent hypoglycemia altogether. According to the American Diabetes Association (ADA), hospitals should adopt a hypoglycemia management protocol that includes a dynamic approach to prevent hypoglycemia from occurring. One example includes whether patients affected could take medications by mouth or intravenously (IV). In response to this situation, a generic protocol was developed by the ADA that successfully utilized continuous monitoring of blood glucose every hour until levels reached 100 mg/dL. Once the patient was stabilized with either a carbohydrate snack or with intravenous dextrose, their blood glucose was rechecked before every meal, at bedtime, and at a set time early in the morning [1]. While prevention is key in this scenario, patients being discharged must be educated of the warning signs of hypoglycemia. They should check with their primary care physician and schedule an appointment one month after being discharged [4]. This idea is to ensure a safe transition from an inpatient setting to a standard outpatient care provider.

Re-education of staff is another fundamental key to helping patients with hypoglycemia. One study showed that re-educating physicians and nurses in the emergency and internal medicine departments through seminars highlighting the ADA’s standards of medical care led to better glycemic control [5]. There were a total of 10 seminars that were 20 minutes in length. As a result, there was a reduction in the use of insulin sliding scales and patients were able to transition to a basal-bolus insulin regimen. The study showed that 65% of patients were able to maintain blood glucose levels between 80 to 180 mg/dL versus only 43% of historical controls. Although the study was not powered to show reductions in mortality or length of hospital stay, the re-education of physicians and nurses in the emergency or internal medicine setting led to the improvement in the glycemic control of their patients [5].

Hypoglycemia has resulted in an increase in mortality and readmission rates in hospitals. Developing a protocol for a glycemic management program in a health-system will allow swift action against the dangers of hypoglycemia. With proper training and education for both medical professionals and patients, hypoglycemia can be closely monitored in the inpatient and outpatient settings to ensure patient safety and overall wellness. 
